# The diagnostic accuracy of circulating free DNA for the detection of KRAS mutation status in colorectal cancer: A meta‐analysis

**DOI:** 10.1002/cam4.1989

**Published:** 2019-02-21

**Authors:** Wenli Xie, Li Xie, Xianrang Song

**Affiliations:** ^1^ Shandong Cancer Hospital Affiliated to Shandong University, Shandong University Shandong Province Jinan P.R. China; ^2^ Shandong Cancer Hospital Affiliated to Shandong University Shandong Academy of Medical Sciences Shandong Province Jinan P.R. China

**Keywords:** cfDNA, colorectal cancer, diagnostic, KRAS mutation, meta‐analysis

## Abstract

KRAS mutations have been reported as a reliable biomarker for epidermal growth factor receptor (EGFR) targeted therapy and are also associated with poor prognosis in colorectal cancer (CRC) patients. However, limitations of detecting KRAS mutations in tissues are obvious. KRAS mutations in the peripheral blood can be detected as an alternative to tissue analysis. The objective of this meta‐analysis was to evaluate the diagnostic value of cfDNA (circulating free DNA) compared with tissues and to investigate the prognostic potential of cfDNA KRAS mutations in CRC patients. Searches were performed in PubMed, Embase, and Cochrane Library for published studies. We extracted true‐positive (TP), false‐positive (FP), false‐negative (FN), true‐negative (TN) values, survival rate of CRC patients with mutant and wild‐type KRAS and calculated pooled sensitivity and specificity, positive/negative likelihood ratios [PLRs/NLRs], diagnostic odds ratios [DORs], and corresponding 95% confidence intervals [95% CIs]. We also generated a summary receiver operating characteristic (SROC) curve to evaluate the overall diagnostic potential. Totally, 31 relevant studies were recruited and used for the meta‐analysis on the efficacy of cfDNA testing in detecting KRAS mutations. The pooled sensitivity, specificity, PLR, NLR, and DOR were 0.637 (95% CI: 0.607‐0.666), 0.943 (95% CI: 0.930‐0.954), 10.024 (95% CI: 6.912‐14.535), 0.347 (95% CI: 0.269‐0.447), and 37.882 (95% CI: 22.473‐63.857), respectively. The area under the SROC curve was 0.9392. Together, the results suggest that detecting KRAS mutations in cfDNA has adequate diagnostic efficacy in terms of specificity. There is a promising role for cfDNA in the detection of KRAS mutations in CRC patients. However, prospective studies with larger patient cohorts are still required before definitive conclusions of the prognostic potential of cfDNA KRAS mutations in CRC patients were drawn.

## 1 INTRODUCTION

Colorectal cancer (CRC) ranks the third in frequency among newly diagnosed cancers worldwide. In 2012, it is the fourth leading cause of death all over the world after lung cancer, liver cancer, and stomach cancer.^1^, ^2^ The prognosis of CRC patients tends to be poor, mainly because most diagnosed CRC cases are at advanced stages. Anti‐epidermal growth factor receptor (EGFR) monoclonal antibodies such as cetuximab and panitumumab have been shown to be effective in metastatic CRC (mCRC) individuals. KRAS mutations have been widely investigated to be a major predictive biomarker for resistance to EGFR blockade in patients with mCRC.^3^ In addition to the most common mutations in codons 12 (Gly12Ala, Gly12Arg, Gly12Asp, Gly12Cys, Gly12Ser, and Gly12Val) and 13 (Gly13Asp) in exon 2, KRAS mutations are also found in exon 3 (codons 59 and 61) and exon 4 (codons 117 and 146) in CRC patients. Patients with mCRC bearing KRAS mutations, are unlikely to benefit from the targeted therapy, showing lower response rates, decreased progression free survival (PFS), and overall survival (OS), compared with the CRC patients with wild‐type KRAS (WTKRAS).^4^, ^5^ Studies have shown that KRAS mutation status is associated with non‐responsiveness,^3^, ^6^, ^7^ indicating that this treatment modality is restricted to patients with WTKRAS. Thus, KRAS oncogene can be used as the most relevant and sensitive molecular biomarker for the responsiveness to EGFR‐targeted therapy in CRC.

Although numerous methods have been developed for determining KRAS mutation status in CRC patients, they are largely dependent on the quality and quantity of tumor tissues^8^ and the data turnaround time is long (2‐3 weeks).^9^ In addition, tumor tissues especially metastatic tumors are rarely available for testing because of practical and ethical reasons.^10^, ^11^ Thus, for determining KRAS mutation status, we need a feasible and sensitive biomarker. Studies have suggested circulating free DNA (cfDNA) as an alternative to tissue analysis for the detection of KRAS mutations.^12^, ^13^ It has reported that cfDNA is an informative, inherently specific, and highly sensitive biomarker for mCRC and high concordance rates exist between cfDNA and tumor tissues.^14^, ^15^, ^16^

Recently, an increasing number of studies utilizing cfDNA for the detection of KRAS mutations in CRC have been reported, but the results turn out to be variable even with some encouraging information. Therefore, we systematically and comprehensively performed the present meta‐analysis to integrate the results of these published studies, aiming to investigate the diagnostic efficiency of cfDNA as a biomarker for KRAS mutations determination compared with the "gold‐standard" tumor tissues and to evaluate the predictive and prognostic value of cfDNA KRAS mutations in CRC patients.

## 2 MATERIALS AND METHODS

### 2.1 Searching strategy

We performed meta‐analysis based on the guidelines in diagnostic studies.^17^ A systematic and comprehensive literature search in PubMed, EMBASE, and The Cochrane Library was performed for potentially relevant and original studies that focused on the diagnostic potential of cfDNA in detecting KRAS mutation in CRC patients. The searching strategy included the combination of following keywords and medical subheadings: “colon cancer” or “colorectal cancer” or “rectal cancer”, “serum” or “plasma” or “circulating”, and "Kras" or "K‐ras". To identify additional studies, reference lists of all relevant publications were also manually screened. No start date limit was applied, and the search ended in December 2017. For a more comparatively overall analysis, we did not set any country restriction, but the included articles must be written in English.

### 2.2 Inclusion and exclusion criteria

Two investigators (Wenli Xie and Xianrang Song) independently inspected the title and abstract of all articles to identify those studies that likely reported the diagnostic potential of cfDNA in detecting KRAS mutations, and then further reviewed full‐text articles to determine whether they were exactly eligible. We included the eligible studies that met the following criteria: (1) all CRC patients were diagnosed by standard test (such as colonoscopy or histopathologic analysis); (2) KRAS mutation status in tumor samples should be determined by cfDNA; (3) sufficient data to reconstruct the diagnostic 2 × 2 contingency table.

We excluded the articles with any of the following characteristics: (1) reviews, comments, letters, and case reports; (2) KRAS mutation status in cfDNA was not compared with tumor samples; (3) duplicate reports from the same patients; (4) insufficient data to construct the diagnostic 2 × 2 table. Agreement between two investigators about each eligible study was reached by the consensus.

### 2.3 Data extraction

The data we extracted from the articles included the first author's name, publication year, country of study, TNM stage, histological type, experimental methods for KRAS mutation detection in cfDNA, serum or plasma, type of tumor tissues, the number of individuals, true positive (TP), false positive (FP), false negative (FN), true negative (TN) values, survival rate of CRC patients with mutant and wild‐type KRAS. When KRAS mutation was determined by multiple methods, we treated it as several separate studies according to the number of methods they used. We conducted data extraction by the same two authors (Wenli Xie and Xianrang Song) independently. Discrepancies between two authors were resolved by the consensus.

### 2.4 Quality assessment

Methodological quality of each eligible articles was assessed by QUADAS‐2 (quality assessment of diagnostic accuracy studies 2) guidelines.^18^ QUADAS‐2 is a tool designed to evaluate the diagnostic accuracy included in the systematic reviews, which consists of four key domains (patient selection, index test, reference standard, and flow and timing). The assessment of the study quality was carried out independently by the same two reviewers (Wenli Xie and Xianrang Song).

### 2.5 Statistical analysis

To analyze the test accuracy, we calculated the pooled sensitivity, specificity, PLR, NLR, positive predicted value, negative predicted value, DOR and corresponding 95% CI after we tabulated TPs, FPs, FNs, and TNs stratified by study. The PLR and NLR were calculated as: sensitivity/(1‐specificity) and (1‐sensitivity)/specificity, respectively. A test was considered of clinical value when a PLR > 5.0 or a NLR < 0.2.^19^, ^20^ DOR, which is calculated as: PLR/NLR, is a measure that combined sensitivity and specificity. Simultaneously, the summary ROC curve (SROC), which based on the sensitivity and specificity of each included study, was generated, and the area under the SROC (AUSROC) was calculated to grade the overall diagnostic accuracy.^21^, ^22^

We used Chi square‐based Q statistic test and the inconsistency index (*I* ^2^) to verify heterogeneity among these studies. Heterogeneity was considered significantly prominent when *I* ^2^ ≥ 50% and *P* ≤ 0.05. In the presence of significant heterogeneity, a random‐effects (DerSimonian‐Laird) model was used to calculate the pooled results. Subgroup analysis was conducted for sample size, countries, detection methods, TNM stages, type of blood samples, storage method of tumor tissues. By virtue of Deek's funnel plot asymmetry test, publication bias was examined.^23^ In addition, meta‐regression analysis was performed to investigate the sources of heterogeneity.^24^

These analyses were undertaken using the STATA software (version 12.0, STATA Corp.) with the MIDAS module and the Meta‐disc. QUADAS‐2 plot was conducted with the Review Manager 5.3. All statistical tests were two‐sided, and a *P* < 0.05 was considered statistically significant.^25^

## 3 RESULTS

### 3.1 Study selection

**Figure 1:**
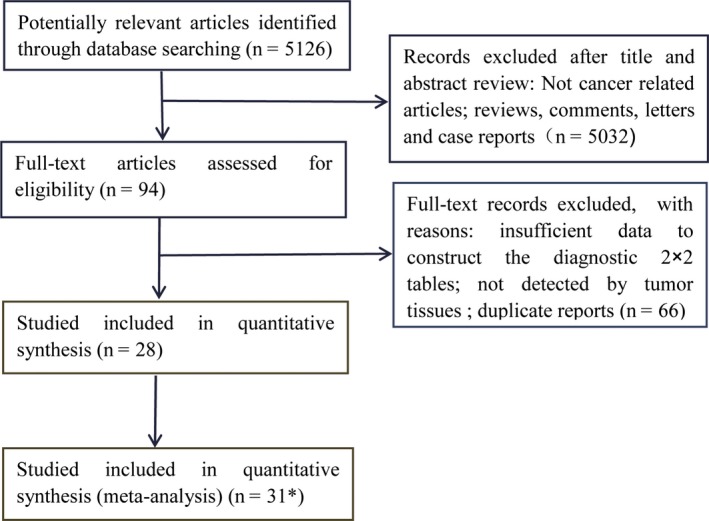
Flow diagram summarizing selection of studies for inclusion in the systematic review. *In the study reported by Taly^47^ and the other study by Xu,^29^ KRAS status was detected by 2 different methods, and Morgan^42^ detected KRAS status both in serum and plasma, and the data from 2 assays and 2 samples were analyzed as 2 independent studies. Thus, 31 eligible studies were included in the meta‐analysis

Flowchart for study selection used in our study is shown in Figure 1. A total of 5126 records were retrieved through our database search. A total of 5032 studies were excluded after primary screening, and 94 articles were selected for further assessment of eligibility. By rigorous evaluation, 28^14^, ^15^, ^16^, ^26^, ^27^, ^28^, ^29^, ^30^, ^31^, ^32^, ^33^, ^34^, ^35^, ^36^, ^37^, ^38^, ^39^, ^40^, ^41^, ^42^, ^43^, ^44^, ^45^, ^46^, ^47^, ^48^, ^49^, ^50^ studies met the inclusion criteria and were included in our present meta‐analysis. In the study reported by Taly^47^ and another study by Xu,^29^ KRAS status was detected by two different methods, and Morgan^42^ detected KRAS status both in serum and plasma, and the data from two different methods and two samples were analyzed as two independent studies. Thus, 31 eligible studies were included in the meta‐analysis. No additionally more relevant articles were recognized after we searched the reference lists of eligible records and related reviews manually.

### 3.2 Characteristics of eligible studies

The main clinical characteristics of 31 eligible studies are presented in Table 1. The 31 eligible articles were published between 2000 and December 2017 and most recruited patients in records were male with metastatic disease. A total of 2565 individuals with CRC were included in this meta‐analysis, and the sample size varied from 12 to 242 individuals with a median size of 65 individuals. Among the 31 studies, 19 used formalin‐fixed paraffin‐embedded (FFPE) tumor tissues to detect KRAS mutation status. The included studies were originated from 12 countries or regions, including Denmark, France, China, Italy, Japan, the USA, Taiwan, Germany, Sweden, England, Ireland, and Korea. Five studies^35^, ^36^, ^48^, ^49^, ^50^ evaluated KRAS status in serum, while the other 26 studies focused on KRAS status in plasma. Various kinds of methods were applied for detecting KRAS mutation in cfDNA, while the amplified refractory mutation system (ARMS) was the most common assay. However, NGS (next‐generation sequencing) and ddPCR (droplet digital PCR） have become more widely used over the last 2 years. In addition, 8 studies investigated the relationship of KRAS mutations in CRC patients with their clinical outcomes. Among them, KRAS mutated patients demonstrated worse OS than those with wild‐type KRAS in 6 studies while a study performed in serum failed to show significant differences in terms of OS. Moreover, Spindler et al^15^ revealed that the higher the mutant plasma KRAS levels are, the worse the PFS and OS of CRC patients was. In the study by Spindler,^28^ mutant KRAS patients had a worse PFS than wild type subjects while there is no significant differences in PFS in 2 other studies by Xu.^29^ QUADAS‐2 summary plot was presented in Figure S1.

**Table 1:** Main characteristics of 31 eligible studies

Author	Year	Country	TNM	Treatment	Sample	Males	AC	Collection^a^	Assays	PFS^b^		OS^b^		N	TP	FP	FN	TN
										Mo (mut vs wt)	*P* Value	Mo (mut vs wt)	*P* Value					
Berger AW	2017	Germany	Metastatic	FFPE	Plasma	NA	NA	Before	ddPCR	—	—	—	—	40	25	2	6	7
Rachiglio AM	2016	Italy	Metastatic	NA	Plasma	57.1%	NA	Before	NGS	—	—	—	—	35	10	0	7	18
Yamada T	2016	Japan	Metastatic	FFPE	Plasma	NA	NA	Before	PNA‐PCR/ddPCR	—	—	—	—	94	34	5	5	50
Beránek M	2016	Czech Republic	Metastatic	FFPE	Plasma	53.1%	NA	NA	NGS	—	—	—	—	32	5	0	1	26
Spindler KL	2015	Denmark	Metastatic	FFPE	Plasma	61.4%	NA	Before	ARMS	—	—	d	<0.05	133	26	4	17	86
Kim ST	2015	Korea	Metastatic	FFPE	Serum	63.1%	NA	Before	RFLP‐PCR	—	—	—	0.991	65	18	8	13	26
Sakai K	2015	Japan	NA	FFPE	Plasma	NA	NA	NA	NGS	**—**	**—**	**—**	**—**	15	5	0	2	8
Danese E	2015	Italy	I‐IV	Frozen	Plasma	65.9%	100%	Before	ARMS	—	—	—	—	85	22	4	5	54
Spindler KL	2015	Denmark	Metastatic	FFPE	Plasma	57%	NA	Before	ARMS	**—**	**—**	**—**	**—**	211	122	3	28	68
Sefrioui D	2015	Japan	Metastatic	FFPE	Plasma	41%	NA	Before After	ddPCR			d	0.04	34	11	0	5	18
Kidness E	2014	USA	I‐IV	Frozen	Plasma	61%	100%	Before	SCODA	**—**	**—**	**—**	**—**	38	15	0	4	19
Xu JM	2014	China	Metastatic	FFPE	Plasma	NA	NA	Before	Direct sequencing	5.4 vs 6.1	0.489	15.7 vs 18.3	0.037	242	30	11	63	138
Xu JM	2014	China	Metastatic	FFPE	Plasma	NA	NA	Before	PNA‐PCR	5.7 vs 6.1	0.274	15.7 vs 19.1	0.009	242	64	12	53	113
Kuo YB	2014	Taiwan	I‐IV	NA	Plasma	53.8%	NA	Before	PNA‐PCR	**—**	**—**	**—**	**—**	52	15	11	0	26
Thierry AR	2014	France	I‐IV	FFPE	Plasma	58.9%	NA	NA	qPCR‐interplex	**—**	**—**	**—**	**—**	95	36	1	3	55
Bettegowda C	2014	USA	Metastatic	NA	Plasma	NA	NA	Before	ddPCR	—	—	—	—	206	68	1	10	127
Perrone F	2014	Italy	NA	FFPE	Plasma	58.3%	100%	Before	ME‐PCR	—	—	—	—	12	0	0	5	7
Taly V	2013	France	Metastatic	Frozen	Plasma	NA	NA	NA	Multiplex dPCR	**—**	**—**	**—**	**—**	50	15	2	4	29
Taly V	2013	France	Metastatic	Frozen	Plasma	NA	NA	NA	Duplex dPCR	**—**	**—**	**—**	**—**	50	17	2	2	29
Spindler KG	2013	Denmark	Metastatic	FFPE	Plasma	65%	NA	After	ARMS	2.7 vs 4.6	0.01	7.8 vs 13.0	<0.001	95	16	1	28	50
Pu XX	2013	China	I‐IV	Frozen	Serum	66.1%	100%	Before	Nested PCR	**—**	**—**	**—**	**—**	115	9	4	28	74
Spindler KL	2012	Denmark	Metastatic	FFPE	Plasma	56%	NA	After	ARMS	^c ^		c		95	32	0	9	54
Liu PJ	2012	China	NA	Frozen	Plasma	NA	NA	Before	COLD‐PCR	—	—	—	—	62	9	4	3	46
Miyano S	2012	Japan	0‐IV	FFPE	Plasma	71.40%	88.1%	Before	PNA‐PCR	—	—	—	—	42	8	2	5	27
Morgan SR	2012	USA	Metastatic	FFPE	Plasma	NA	NA	After	ARMS	—	—	—	—	71	8	0	24	39
Morgan SR	2012	USA	Metastatic	FFPE	Serum	NA	NA	After	ARMS	—	—	—	—	71	5	0	27	39
Lefebure B	2010	France	Metastatic	FFPE Frozen	Serum	61.3%	100%	NA	PNA‐PCR	—	—	—	—	23	7	0	7	9
Trevisiol C	2006	Italy	I‐IV	Frozen	Serum	53%	NA	NA	ME‐PCR	**—**	**—**	d	0.02	86	10	1	18	57
Lindforss U	2005	Sweden	I‐IV	FFPE	Plasma	36%	NA	NA	TGGE	**—**	**—**	**—**	**—**	25	9	0	7	9
Mulcahy HE	2000	England	NA	Frozen	Plasma	71.40%	NA	Before	MASA‐PCR	**—**	**—**	**—**	**—**	14	6	0	1	**7**
Kopreski MS	2000	USA	NA	FFPE	Plasma	NA	NA	Before	RFLP‐PCR	—	—	—	—	135	29	7	6	93

AC, adenocarcinoma; ARMS, Scorpion Amplification Refractory Mutation System; ddPCR, droplet digital PCR; HRM, high‐resolution melting; MASA, mutant allele‐specific amplification; ME‐PCR, mutant‐enriched PCR; Mo (mut vs wt): median survival (month, mutant KRAS patients vs wild type subjects); N, number; NA, not available; NGS, next‐generation sequencing; RFLP‐PCR, restriction fragment length polymorphism PCR; OS: overall survival; PFS: progression‐free survival; PNA, Peptide Nucleic Acid (PNA)‐mediated PCR clamping; SCODA, sequence‐specific synchronous coefficient of drag alteration; TGGE, temperature gradient gel electrophoresis. ^a^Collection time of blood samples; ^b^plasma/serum KRAS status; ^c^Worse PFS and OS of patients with high levels of mutant plasma KRAS, data not shown in the study; ^d^Worse OS of patients with mutant KRAS.

### 3.3 Accuracy of cfDNA for the detection of KRAS mutations

**Figure 2:**
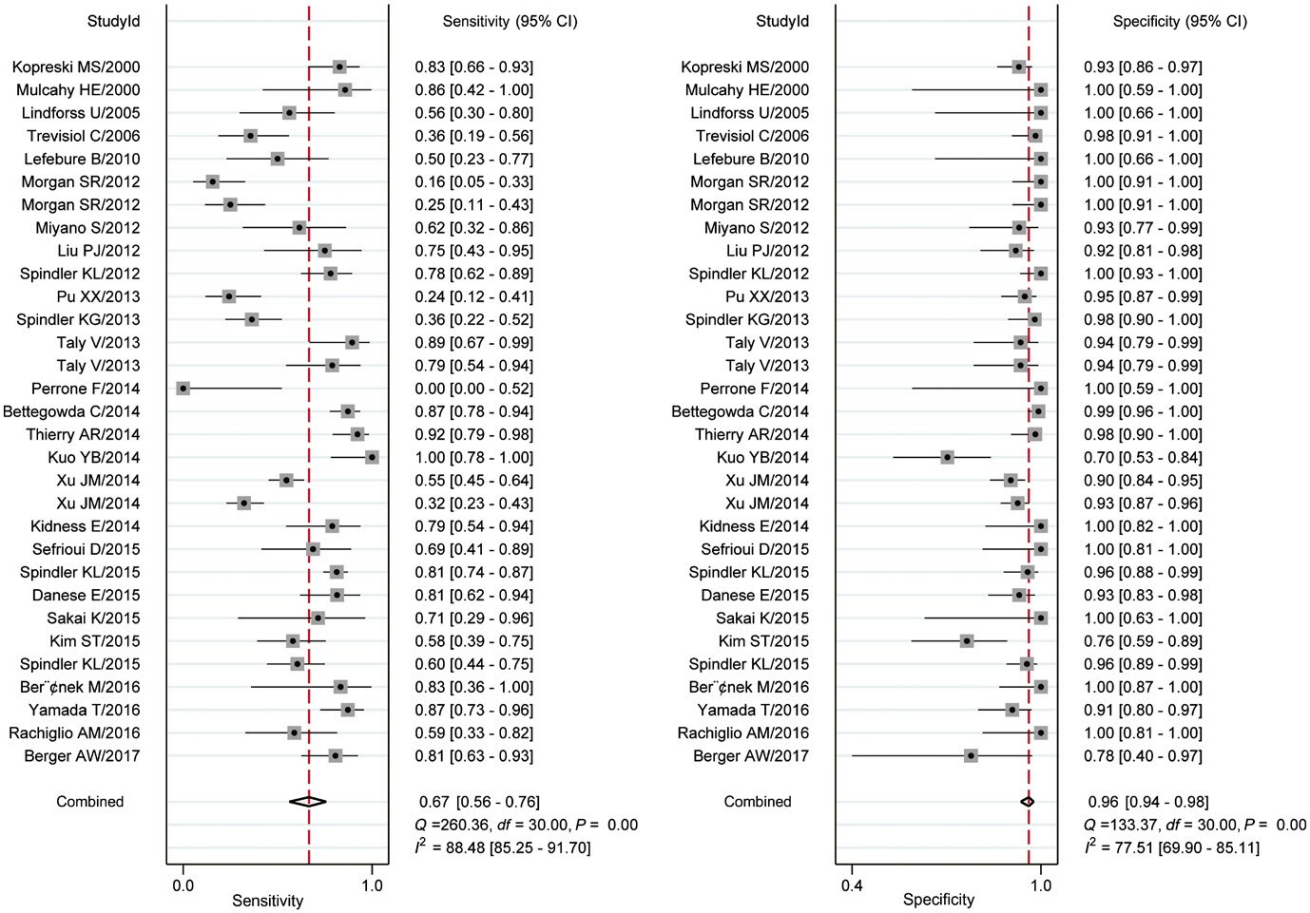
Forest plots of the sensitivity and specificity of circulating free DNA (cfDNA). The pooled sensitivity was 0.637 (95% confidence intervals [95% CIs]: 0.607‐0.666), and the pooled specificity was 0.943 (95% CI: 0.930‐0.954)

**Figure 3:**
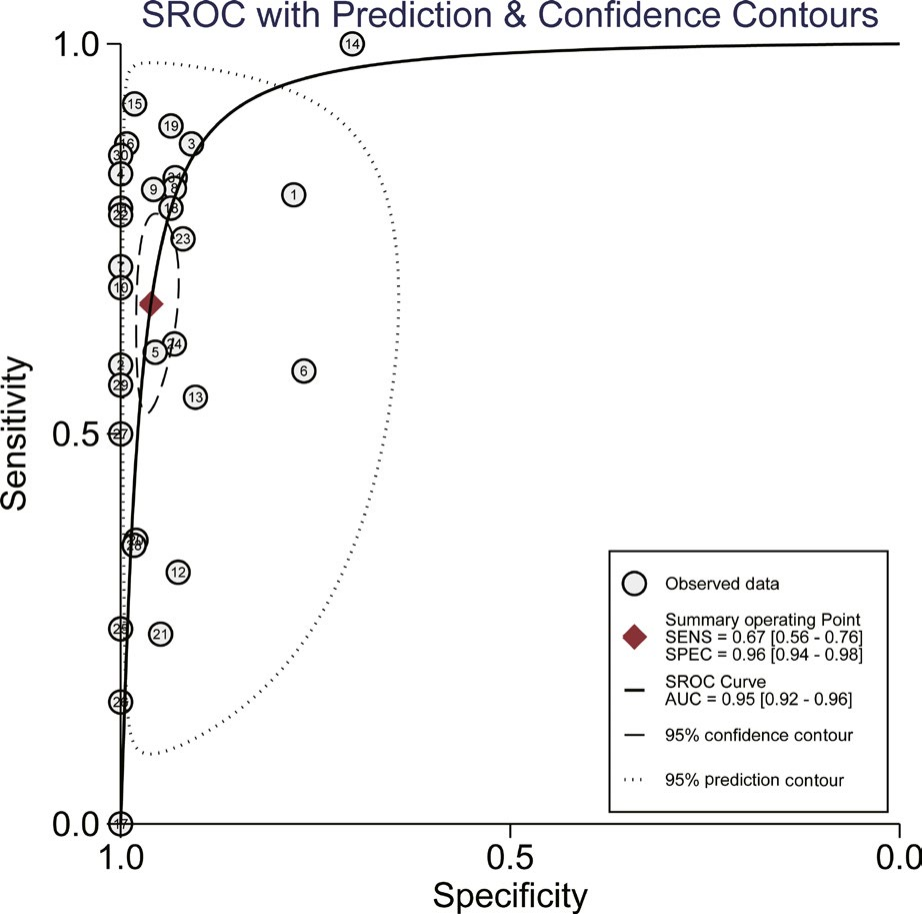
Summary Receiver Operating Characteristic (SROC) Curve for circulating free DNA (cfDNA) on detection of KRAS status among colorectal cancer (CRC) patients in all studies. The figure also shows 95% confidence contour and 95% prediction contour

**Figure 4:**
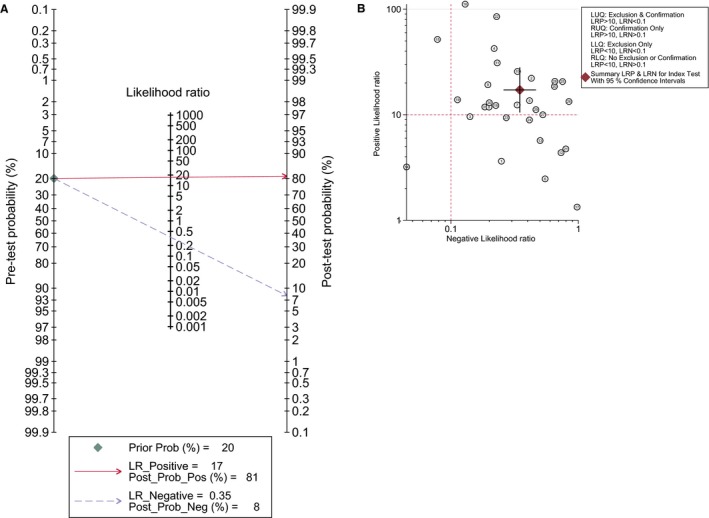
Fagan nomogram presents the clinical utility of circulating free DNA (cfDNA) for the detection of KRAS mutations (A). The likelihood ratio matrix of cfDNA for the detection of KRAS mutation (B)

The pooled sensitivity and specificity calculated by the bivariate random effects model were 0.637 (95% confidence intervals [95% CI: 0.607‐0.666]) and 0.943 (95% CI: 0.930‐0.954), respectively (Figure 2). The overall positive likelihood ratio (PLR) and negative likelihood ratio (NLR) were 10.024 (95% CI: 6.912‐14.535) and 0.347 (95% CI: 0.269‐0.447), respectively (Figure S2). The diagnostic odds ratio (DOR) was 37.882 (95% CI: 22.473‐63.857). Figure 3 was the SROC curve of cfDNA and the AUSROC was 0.9392. We also constructed fagan plot (Figure 4A) and the likelihood ratio scatter matrix to present the diagnostic potential of cfDNA visually (Figure 4B).

### 3.4 Threshold effect and heterogeneity

In diagnostic studies, one of the major sources of between‐study heterogeneity is the threshold effect. The forest plots of sensitivity, specificity, PLR, NLR, and DOR indicate that between‐study heterogeneity is significantly prominent*.* We used 2 different methods to assess if the threshold effect contributed to the heterogeneity. Visual evaluation of ROC plane revealed the absence of significant threshold effect as there was not a typical pattern of "shoulder arm" (Figure S3). Additionally, the Spearman correlation coefficient (0.224, *P* = 0.233) was calculated, further confirmed no significant threshold effect. Thus, we performed meta‐regression and subgroup analysis to explore the potential sources of heterogeneity, 7 covariates were used in our present meta‐regression: including sample size, countries, detection methods, and TNM stages, type of blood samples, collection time of blood samples, storage method of tumor tissues. The results revealed that between‐study heterogeneity was not correlated with other covariates except type of blood samples (*P* = 0.0225).

### 3.5 Subgroup analysis

Subgroup analysis was conducted to investigate the influence of potential confounding factors, including countries, sample size, TNM stages, detection methods, and type of blood samples, collection time of blood samples, storage method of tumor tissues. Results were shown in Table 2. Both the AUSROC and DOR were higher in plasma samples, in fresh frozen tissues and in large size studies compared with that in serum samples, in FFPE tissues and in small size studies, respectively. However, the AUSROC was higher in CRC patients with I‐IV and in blood samples which were collected after chemotherapy than those with metastasis and in blood samples collected before chemotherapy, respectively. ARMS showed the highest AUSROC and DOR value among all the assays assessed.

**Table 2:** Meta‐analysis results

	Studies	AUSROC	Sensitivity	Specificity	PLR	NLP	DOR
Overall	31	0.9392	0.637 (0.607‐0.666)	0.943 (0.930‐0.954)	10.024 (6.912‐14.535)	0.347 (0.269‐0.447)	37.882 (22.473‐63.857)
Country							
USA	5	0.9812	0.638 (0.566‐0.705)	0.975 (0.952‐0.989)	23.503 (7.075‐78.077)	0.333 (0.123‐0.901)	91.255 (24.281‐342.96)
Denmark	4	0.9690	0.705 (0.638‐0.758)	0.970 (0.942‐0.987)	17.828 (9.115‐34.869)	0.336 (0.171‐0.658)	59.551 (24.419‐145.23)
France	4	0.9834	0.824 (0.730‐0.896)	0.961 (0.911‐0.987)	16.266 (7.163‐36.940)	0.191 (0.059‐0.618)	104.82 (28.110‐390.84)
China	4	0.9262	0.432 (0.371‐0.459)	0.923 (0.892‐0.947)	5.477 (3.794‐7.907)	0.626 (0.469‐0.836)	9.208 (5.065‐16.740)
Italy	4	0.9534	0.583 (0.461‐0.698)	0.963 (0.915‐0.988)	13.757 (6.011‐31.482)	0.408 (0.196‐0.851)	49.406 (16.619‐146.88)
Japan	4	0.9524	0.773 (0.662‐0.862)	0.936 (0.873‐0.974)	10.169 (5.162‐20.033)	0.289 (0.176‐0.473)	47.564 (18.249‐123.97)
Sample size							
Small	24	0.9325	0.645 (0.602‐0.686)	0.942 (0.923‐0.958)	10.441 (6.447‐16.912)	0.334 (0.242‐0.462)	40.997 (23.990‐70.062)
Large	7	0.9757	0.629 (0.588‐0.670)	0.943 (0.924‐0.959)	9.784 (5.222‐18.338)	0.361 (0.220‐0.592)	29.925 (10.418‐85.959)
Format of blood samples							
Plasma	26	0.9464	0.681 (0.650‐0.711)	0.943 (0.929‐0.955)	11.127 (7.481‐16.549)	0.289 (0.214‐0.391)	49.796 (28.452‐87.150)
Serum	5	0.7032	0.345 (0.267‐0.429)	0.940 (0.900‐0.968)	5.084 (2.171‐11.907)	0.720 (0.603‐0.860)	7.169 (3.511‐14.640)
TNM stage							
Metastatic	18	0.9216	0.624 (0.590‐0.657)	0.948 (0.932‐0.961)	10.519 (6.356‐17.408)	0.370 (0.273‐0.502)	35.955 (17.789‐72.672)
I‐IV	8	0.9597	0.639 (0.567‐0.707)	0.933 (0.901‐0.957)	10.103 (4.097‐24.913)	0.325 (0.174‐0.605)	41.587 (13.996‐123.57)
Storage method of tumor tissues							
FFPE	19	0.9220	0.615 (0.580‐0.649)	0.942 (0.925‐0.956)	9.304 (5.997‐14.434)	0.376 (0.280‐0.506)	31.517 (16.377‐60.655)
Frozen	8	0.9669	0.613 (0.535‐0.687)	0.949 (0.919‐0.970)	10.375 (6.478‐16.618)	0.308 (0.168‐0.564)	36.563 (16.044‐83.324)
Collection time of blood samples							
BC	18	0.9293	0.668 (0.633‐0.701)	0.926 (0.909‐0.941)	7.853 (5.144‐11.987)	0.312 (0.219‐0.445)	30.030 (15.617‐57.745)
AC	4	0.9987	0.409 (0.330‐0.493)	0.995 (0.970‐1.000)	24.332 (6.935‐85.370)	0.597 (0.396‐0.900)	42.708 (11.603‐157.20)
Detection methods							
ARMS	7	0.9638	0.626 (0.574‐0.676)	0.970 (0.948‐0.984)	15.655 (9.208‐26.617)	0.476 (0.346‐0.655)	52.952 (28.078‐99.861)
PNA‐PCR	4	0.9038	0.591 (0.511‐0.668)	0.875 (0.821‐0.917)	4.718 (2.807‐7.931)	0.272 (0.126‐0.590)	13.631 (7.319‐25.386)
ddPCR	3	0.8757	0.832 (0.755‐0.893)	0.981 (0.944‐0.996)	20.112 (1.403‐288.379)	0.214 (0.112‐0.412)	97.242 (6.427‐1471.3)
NGS	3	0.9268	0.667 (0.472‐0.827)	1.000 (0.932‐1.000)	22.531 (4.588‐110.647)	0.379 (0.239‐0.601)	69.376 (11.199‐429.77)
RFLP‐PCR	2	0.500	0.712 (0.587‐0.817)	0.888 (0.822‐0.936)	5.371 (1.147‐25.145)	0.328 (0.097‐1.111)	16.812 (1.240‐228.03)

Data were present as accuracy data with 95% confidence intervals. AC, after chemotherapy; AUSROC, area under curve; BC, before chemotherapy; DOR, diagnostic odds ratio; NLR, negative likelihood ratio; PLR, positive likelihood ratio.

### 3.6 Sensitivity analysis and publication bias

**Figure 5:**
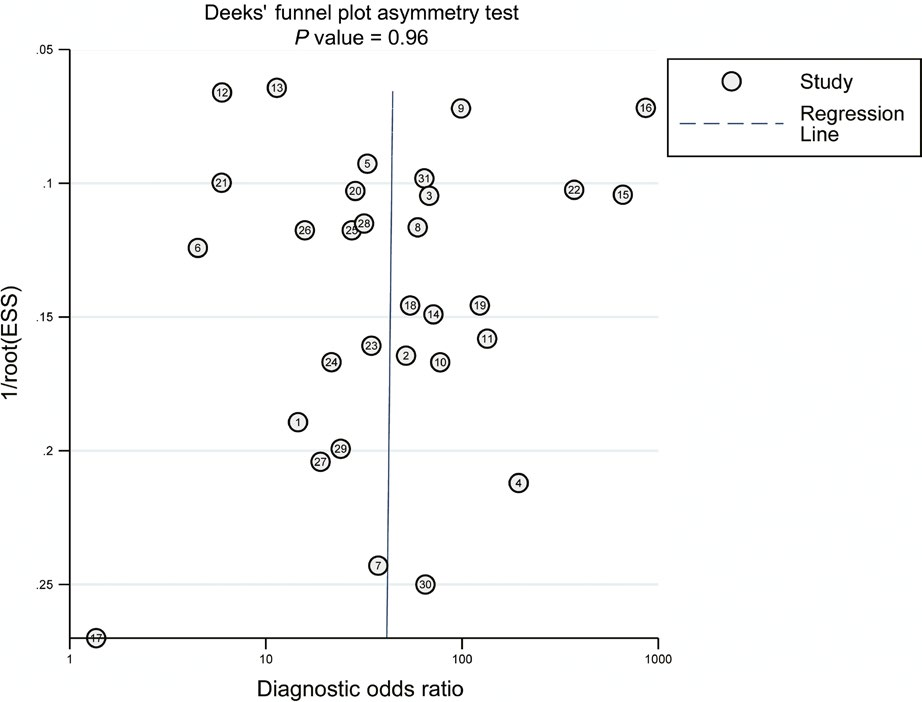
Assessment of the potential publication bias of the 31 included studies. The *P* value of Deek's funnel plot was 0.96, suggesting no significant publication bias

**Figure 6:**
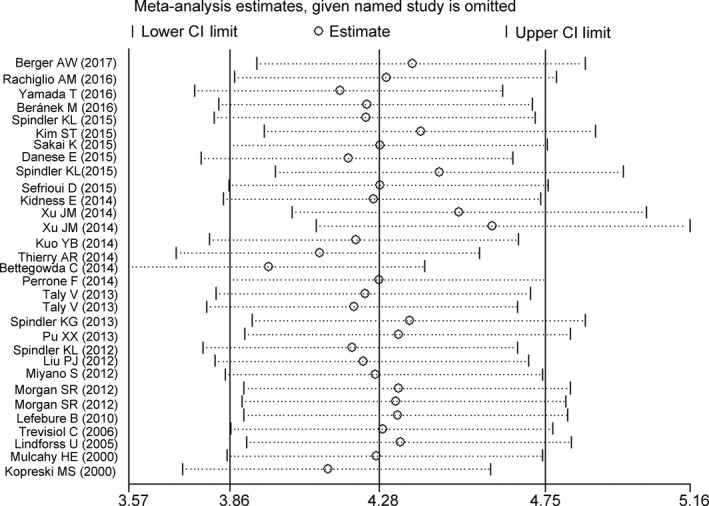
Sensitivity analysis of the 31 eligible studies. The results indicated that the pooled results were robust and not influenced by individual studies

Publication bias of the included studies was examined by Deek's funnel plot asymmetry test. A *P* value of 0.96（>0.05) suggested no obvious publication bias (Figure 5). Results of our sensitivity analysis revealed that the pooled estimate was robust (Figure 6).

## 4 DISCUSSION

Personalized targeted therapy is based on certain gene mutation status of primary tumors， such as EGFR and KRAS. As obvious limitations exist while detecting gene mutations in tumor tissues, cfDNA has attracted more and more attention due to its more feasible, timely, and less invasive nature.^51^, ^52^ For example, in pancreatic cancer, KRAS mutations in cfDNA were evaluated using deep sequencing assay as non‐invasive biomarkers in a large case‐control study and its changes between preoperation and postoperation were suggested to be a predictive biomarker for survival and treatment response.^53^, ^54^ Currently, the KRAS status of primary tumors in CRC patients is considered as a reliable selection criterion prior to the application of EGFR‐targeted therapy**. **During the past decades, relevant studies have been performed to assess the diagnostic potential, and predictive and prognostic value of cfDNA for the determination of KRAS status in CRC patients. Comparisons between the various studies are difficult to make, not at least due to a lack of standardized procedures and technologies for isolation and quantification of cfDNA. Other factors, such as format of blood samples, storage method of tumor tissues, and detection methods of KRAS mutations, also matter the limited generalizability. Hence, we performed a comprehensive meta‐analysis on the potential and predictive and prognostic value of cfDNA in detecting KRAS status in CRC patients.

We included 31 studies with a total of 2565 participants. Calculated with the bivariate random effects model, the detection of KRAS status using cfDNA in CRC patients yielded a pooled sensitivity of 63.7% and an overall specificity of 94.3%. The AUSROC, which represents a global summary measure of the SROC curve, is regarded as an overall measure of diagnostic value. In this meta‐analysis, the AUSROC was 0.9392, indicating a high level of diagnostic accuracy. Of note, the value of DOR, which is a single indicator of diagnostic test performance,^55^ was 37.882 in our study, revealing a high level of overall accuracy.

Likelihood ratios and posttest probabilities, 2 parameters used for assessing clinical utility of the diagnostic test,^19^ are also important for a biomarker. They presented us the likelihood whether a patient with positive or negative result has KRAS mutation. Compare with prior probability (20%), the positive posttest probability (81%) is much higher and the negative posttest probability is quite lower (<0.1), indicating a high diagnostic potential of cfDNA in detecting KRAS status in CRC patients (Figure 4A)*. *As shown in Figure 4B, cfDNA is located in the right upper quadrant (PLR > 10, NLR > 0.1), demonstrating that cfDNA could act as a test to confirm KRAS mutation.

cfDNA showed high diagnostic accuracy in our meta‐analysis; however, high statistical heterogeneity among studies existed simultaneously. To explore the possible sources of heterogeneity, we performed meta‐regression. The results of the regression showed that the sample type was the statistically significant variable that contributed to the heterogeneity. In addition, subgroup analyses provided the information whether these factors (sample size, countries, detection methods, and TNM stages, type of blood samples, collection time of blood samples, storage method of tumor tissues) could affect the diagnostic accuracy of cfDNA. Indeed, as previously reported in the published researches,^42^, ^56^ the outcomes of stratified analysis demonstrated that cfDNA from plasma had higher diagnostic accuracy than that from serum, suggesting that cfDNA in plasma may perform better in detecting KRAS status in CRC individuals.

Many methods have been developed to determine KRAS mutations in cfDNA, such as direct sequencing, the scorpion‐ARMS, peptide nucleic acid‐mediated polymerase chain reaction (PNA‐PCR) clamping method, next‐generation sequencing (NGS), ddPCR, etc. Moreover, with the development of test assays, the diagnostic accuracy of cfDNA has been improved. ARMS and PNA‐PCR are the 2 most frequently used methods in clinical practice and several detection kits based on ARMS and PNA‐PCR are available commercially. Moreover, over the last 2 years, NGS and ddPCR have been widely used. Our subgroup analysis confirmed that the one with the highest overall diagnostic performance was ARMS according to the AUSROC (0.9638), which showed great potential to be the most promising approach, while it was ddPCR by the value of DOR (97.242). We also revealed that the method with the highest sensitivity and the highest specificity among all the assays assessed was ddPCR and NGS, respectively. We did not perform subgroup analysis for other test methods due to limited studies.

Usually, CRC patients at advanced stage have higher levels of circulating‐free DNA than those at the early stage. Furthermore, studied have demonstrated that cfDNA is expected to be negative if the proportion of cfDNA in a sample is lower than 0.01%.^12^, ^57^ Hence, we could conclude that the detection rates of cfDNA would be higher in patients with advanced stage, whose cfDNA was much greater by contrast. Although the clear mechanism that determines the release of cfDNA is indefinite until now, present hypotheses suggest that apart from tumor size, cfDNA level is related to another factor, site of metastasis. For example, in CRC patients, the amount of cfDNA is significantly higher when the metastasis appears in the liver than that in the lung*.* 26 TNM stage acts as an appropriate indicator that combines tumor volume and metastasis. However, subgroup analysis found that AUSROC was higher in CRC individuals with I‐IV than those with metastasis; this might be explained by the fact that few studies were available for CRC patients at an early stage.

To date, the most frequently used tissue samples were FFPE tissues, which might lead to significant DNA degradation and lead to FP or FN results. However, liquid nitrogen frozen tissues do not have the problem. Our stratified analysis revealed that both the AUSROC and DOR values were higher in frozen tissues, confirming that the diagnostic accuracy of cfDNA performed better in the frozen tissues than FFPE tissues.

Generally, if studies involved more participants, the diagnostic performance of cfDNA would be higher. And our subgroup analysis confirmed that the concordance rate was higher in studies with large size (>100). The AUSROC in large size studies (0.9757) was higher compared with that in small size studies (0.9325).

In addition, studies have demonstrated that chemotherapy could influence KRAS mutation status, thus, the timing of tissue collection and peripheral blood collection matter the concordance rate. As measured by AUSROC, cfDNA had higher diagnostic accuracy when blood sample was collected after chemotherapy; this might be due to the fact that only few studies in which blood samples were collected after chemotherapy were available.

In this meta‐analysis, both the pooled sensitivity (0.637) and the overall specificity (0.943) for cfDNA were high, but it turned out that the specificity was much higher. Studies have pointed out that the key advantage of cfDNA is the high degree of specificity,^52^ since other methods used for determining sensitive KRAS mutation status, such as biopsy, usually trigger invasive diagnostic procedures. Both the fagan nomogram and likelihood ration scattergram demonstrated that cfDNA might serve as a suitable screening test to recognize the individuals with KRAS mutations. The high specificity of cfDNA, together with the non‐invasive nature, make real‐time monitoring of KRAS status during treatment of targeted chemotherapy of CRC patients possible.^58^, ^59^

Of note, in addition to methodological issues, the intratumor heterogeneity also matters the concordance rate of KRAS mutations between cfDNA and tumor tissues, with different areas of the same tumor showing different genetic profiles. According to our study, the discordance of KRAS mutations between tissue and blood may be addressed in a large measurement by using plasma samples rather than serum samples, by utilizing approach with high diagnostic potential (e.g., ARMS), as well as by using fresh frozen tissues rather than FFPE tissues.

KRAS mutations have been shown to be associated with poor prognosis in cancer patients, including CRC and KRAS status is an independent prognostic factor for OS and PFS.^54^, ^60^ cfDNA KRAS mutations are also reported to be predictors of poor prognosis for mCRC.^36^ So far, 7 out of 8 studies revealed poor prognostic potential of KRAS mutations in blood in terms of OS. However, a study performed in serum and other 2 studies in plasma failed to show significant differences in terms of OS and PFS, respectively. We should note that one of the studies showing no significance was performed in serum, in which diagnostic potential of cfDNA was relatively low compared with plasma, as was shown in our results in Table 2. However, whether these discrepancies are due to methodological limitations or to cfDNA biology should be further assessed. After all, multiple gene mutations lead to the tumorigenesis and progression of CRC and may affect the prognosis of CRC patients, as well as KRAS.^61^, ^62^ Also, the number of studies investigating the prognostic potential of KRAS mutations in CRC in cfDNA was small. Therefore, prospective studies in large patient cohorts are still required before making definitive conclusions for the prognostic potential of cfDNA KRAS mutations in CRC patients. The conclusions were consistent with those in lung cancer.^63^

As a review research assessing the diagnostic potential and predictive and prognostic value of cfDNA for the detection of KRAS mutation status, the results of our study are promising and may act as an attempt to provide guidance for future studies. However, several limitations in this study should be pointed out. First, the other activating mutations of the RAS family such as NRAS, which may also confer resistance to EGFR blockade in patients without KRAS mutations,^64^ have not been integrated in our study**. **Further studies are needed to clearly identify the diagnostic value and prognostic and predictive potential of NRAS in cfDNA in CRC patients. Second, subgroup and sensitivity analysis were conducted to assess the influences of small‐sized studies, although results confirmed that the pooled results were robust and not affected by bias, we also should recognize that the number of included studies was relatively small for several stratified analyses, which may easily lead to bias. So we should be in caution when we interpreted the outcomes of the subgroup analysis. Third, substantial heterogeneity was observed. Threshold effect did not contribute to the heterogeneity after we performed the ROC plane and calculated the Spearman correlation coefficient. Results of the meta‐regression showed that no other analyzed factors could account for the majority of heterogeneity except the sample type. Our study also revealed that plasma can act as a more promising matrix for detecting KRAS mutation status than serum. It is worth noting that apart from the characteristics analyzed, many other factors, such as ethnicity, percentage of CRC adenocarcinoma, methodologic quality, were not included because of the unavailable adequate information used for analysis, which might be the potential sources of heterogeneity. In addition, although high impact reports tend to be published in English in PubMed and EMBASE, the 2 most comprehensive medicine databases, it was still possible that some non‐English studies were not included in this meta‐analysis. Furthermore, numerous QUADAS items could only be judged as “unclear”, indicating that bias may exist in patient selection and the interpretation of reference and index tests. Moreover, the publication bias of included studies was examined by Deek's funnel plot asymmetry test. A P value of 0.96 indicated no obvious publication bias.

In conclusion, cfDNA could serve as an effective method to detect KRAS mutation status in CRC. Due to its non‐invasive nature, cfDNA might be a promising screening tool for CRC patients.

## CONFLICT OF INTEREST

None declared.
