# Supplementary figures and images for: The diagnostic accuracy of circulating free DNA for the detection of KRAS mutation status in colorectal cancer: A meta‐analysis
Source: Cancer Med. 2019 Feb 21;8(3):1218–31. doi: 10.1002/cam4.1989 (PMC6434340; doi:10.1002/cam4.1989)

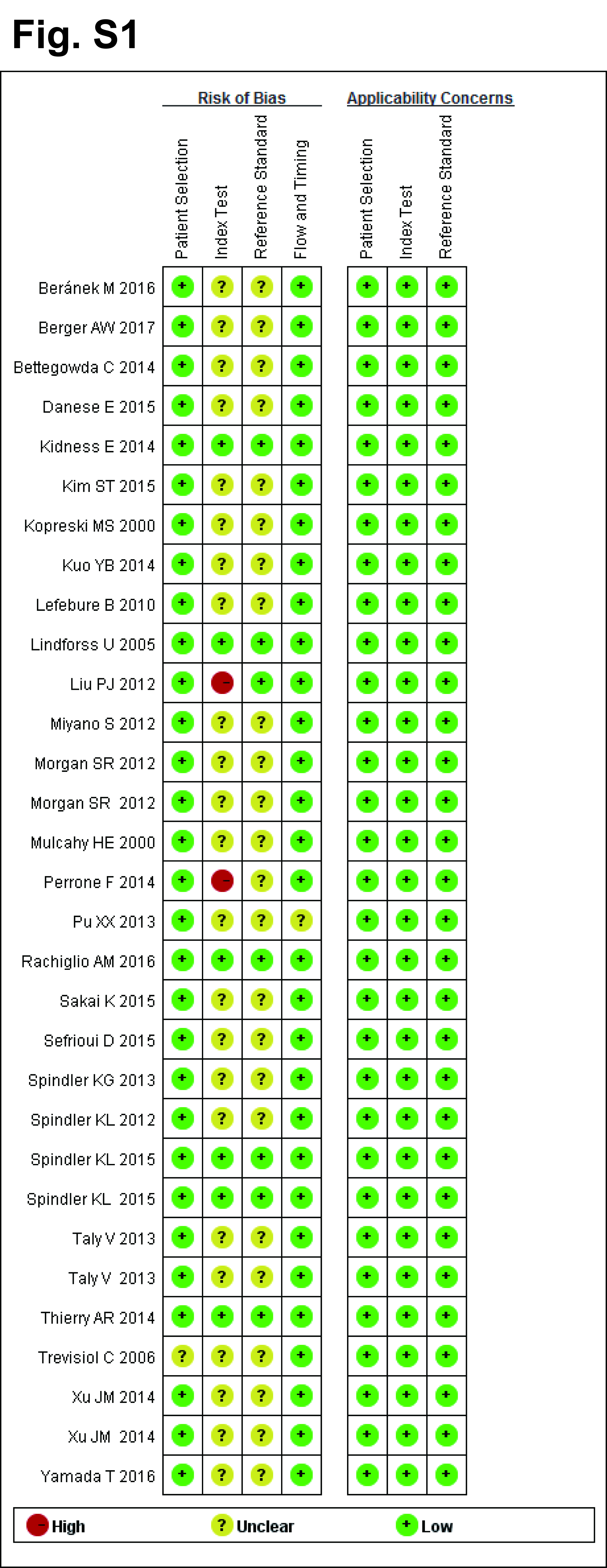

Supplement: Supplementary file 1 [file CAM4-8-1218-s001.tif]

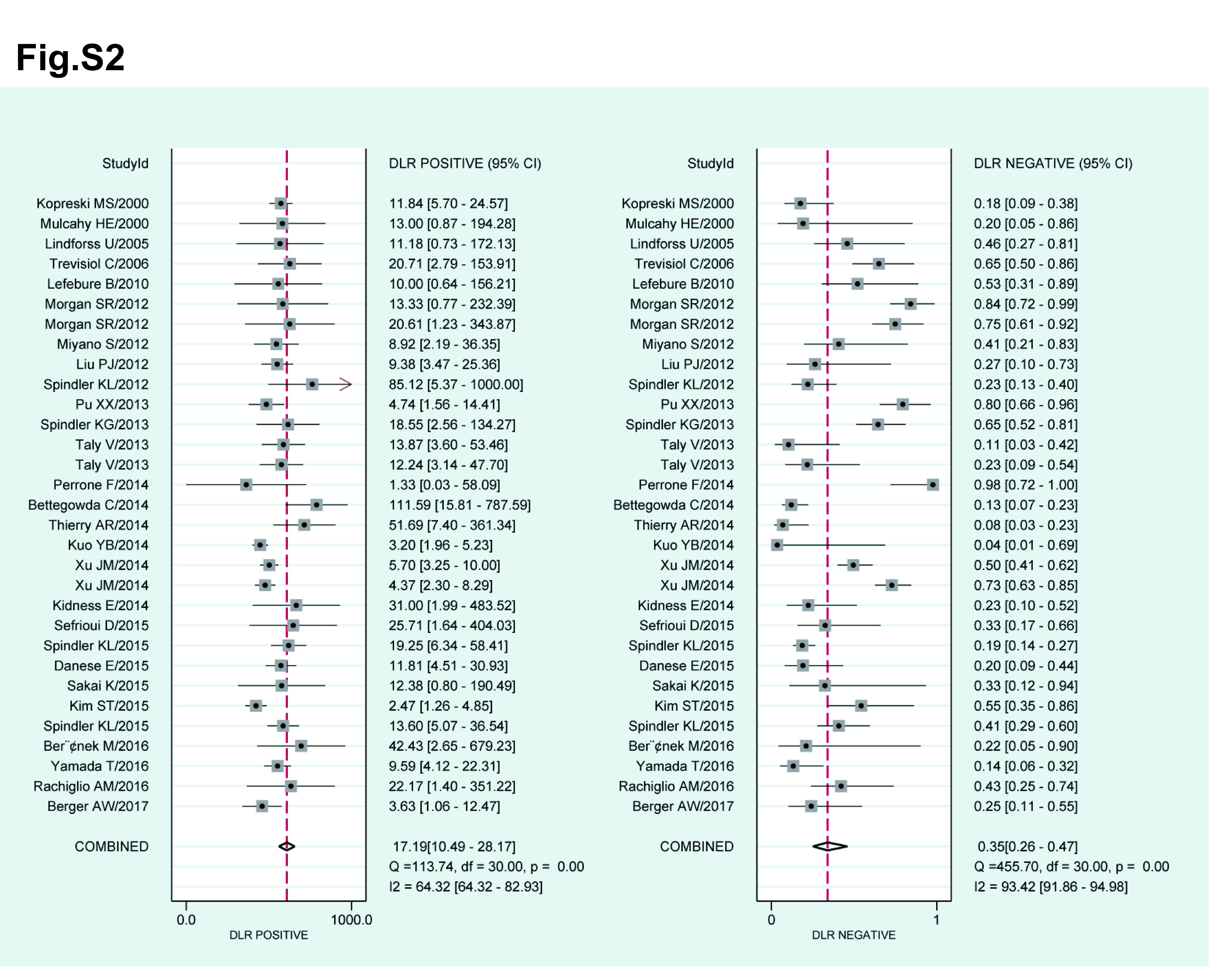

Supplement: Supplementary file 2 [file CAM4-8-1218-s002.tif]

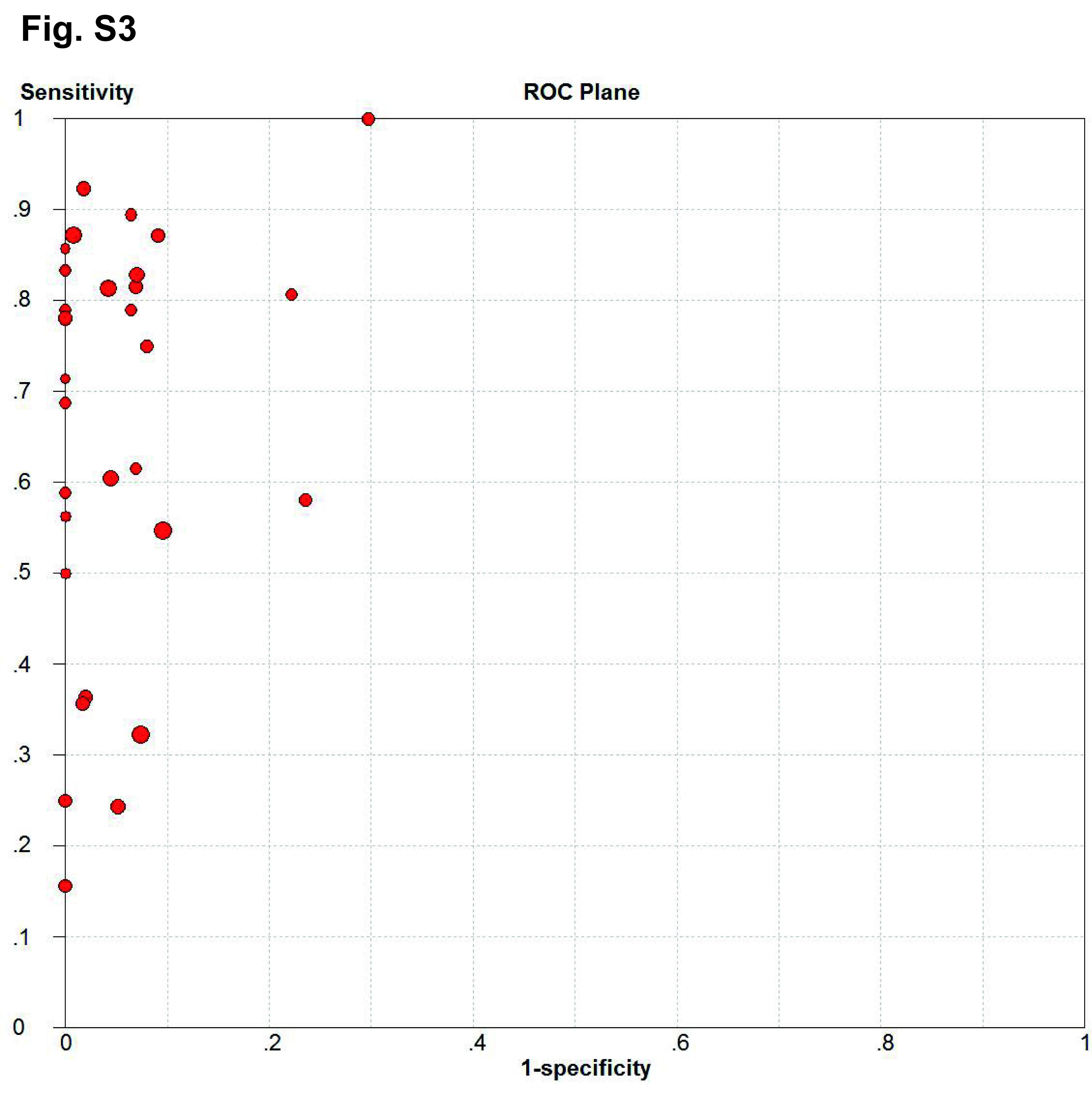

Supplement: Supplementary file 3 [file CAM4-8-1218-s003.tif]
